# Development of an Anti-Immunocomplex Antibody and Non-competitive Immunoassay for the Detection of Testosterone

**DOI:** 10.1021/acs.analchem.5c06003

**Published:** 2026-02-26

**Authors:** Ida Bäckström, Urpo Lamminmäki, Etvi Juntunen, Janne Leivo

**Affiliations:** † Department of Life Technologies and InFLAMES Flagship, 8058 University of Turku, Turku 20014, Finland; ‡ Olo Health Oy, Turku 20100, Finland; § FICAN West Cancer Centre Laboratory, University of Turku and Turku University Hospital, Turku 20014, Finland

## Abstract

Accurate and sensitive quantification of testosterone using simple, high-throughput, and low-cost methods has remained a longstanding analytical challenge, despite the issue being highlighted by the endocrinology community more than a decade ago. Existing competitive immunoassays lack sensitivity and specificity, while reference methods based on mass spectrometry are complex, costly, and unsuitable for routine large-scale testing. Alternative approaches are needed to aid in the diagnosis of androgen deficiency and excess in men, women, and children. In this study, we report a novel anti-immunocomplex (anti-IC) antibody that recognizes a monoclonal anti-testosterone antibody bound to testosterone. The anti-IC antibody was generated using phage display, and the antibody pair was utilized in the development of a non-competitive time-resolved fluorescence immunoassay for the detection of testosterone. The assay demonstrated reliable detection within the physiological range of testosterone in men, women, and children and compatibility with plasma as a sample matrix. These findings emphasize the potential of novel anti-IC antibodies in the development of more sensitive immunoassays, offering accessible alternatives to existing methods for testosterone analysis.

## Graphical abstract

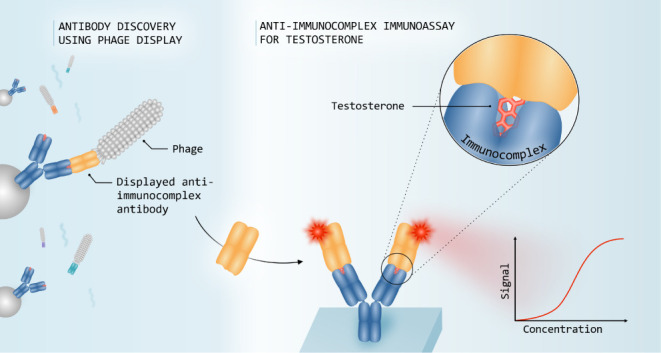

As one of the primary androgens, testosterone plays a crucial role in regulating reproductive physiology and sexual differentiation. Substantial advancements have enhanced our understanding of its role in both health and disease, extending its relevance to conditions such as polycystic ovary syndrome (PCOS), hypogonadism, osteoporosis, cancer, diabetes, and cardiovascular disease.^1^ As the population ages and the prevalence of obesity rises, the risk for male hypogonadism increases.^2,3^ The sharp increase in testosterone prescriptions between 2000 and 2011 not only reflects this trend, but also underscores the growing need for accurate testosterone assays to diagnose hypogonadism.^4^ In addition, accurate measurement of testosterone across a wide concentration range is essential to diagnose androgen deficiency and excess in women, as expressed in conditions such as PCOS and adrenal and ovarian tumors.^5,6^

Liquid chromatography-tandem mass spectrometry (LC-MS/MS) is considered the gold standard method for quantifying testosterone in blood,^7,8^ and while it is a highly sensitive method, it is costly and time-consuming.^9^ Immunoassays offer several advantages over MS-based techniques, including high throughput, operational simplicity, rapid detection, and lower cost.^3,9^ Direct immunoassays, which measure the analyte directly from the sample without pre-extraction, offer high precision and throughput but lack the accuracy needed for detecting low testosterone concentrations. Following analyses and comparisons of 16 commercially available direct testosterone immunoassays performed by Taieb et al. (2003) and Wang et al. (2004),^10,11^ The Endocrine Society issued a position statement in 2007 advising against the use of direct immunoassays for measuring testosterone.^12^ In light of the position statement calling for improved testosterone assays and the subsequent lack of published literature in this area, it is clear that alternative solutions or innovations to address this issue are still urgently needed.

A major limitation of current small-molecule immunoassays is their insufficient sensitivity, as they are almost exclusively performed in a competitive format.^13^ Testosterone and other haptens have posed a challenge in immunoassay development because of their small molecular size, which limits their surface area and prevents the use of conventional, and generally more sensitive, non-competitive immunoassay formats such as the sandwich assay. The signal readout in the non-competitive assay is directly proportional to the analyte concentration, meaning that the more analyte there is in the sample, the more the signal increases. This contributes to the sensitivity of the assay, as low concentrations of analyte can be observed as a value above zero. Competitive assays, on the other hand, have an inversely proportional readout, making it difficult to establish the exact threshold (limit of detection, LoD) at the high end of the signal curve under which analyte concentrations can be distinguished.^14,15^

Novel immunoassay concepts have been developed for small molecules as alternatives to the traditional assays. These include the idiometric assay,^16^ the open-sandwich ELISA (OS-ELISA),^17^ and the anti-immunocomplex (anti-IC) assay^18^—all of which have been successfully applied in the detection of other hormones, such as estradiol. The anti-IC assay is done in a non-competitive format where the IC is formed by the primary antibody and its small analyte, consecutively recognized by a secondary anti-IC antibody. In addition to increased sensitivity, the use of two antibodies in the anti-IC assay reduces the risk of cross-reactivity by structurally similar compounds^19^ and eliminates the need for analyte labeling. The non-competitive format also allows for the use of excess reagents, which can enhance the assay kinetics and further support the development of robust immunoassays. In addition, in comparison with the competitive immunoassay formats where the analytical performance is solely dependent on the binding kinetics of the primary antibody, the anti-IC antibodies do not require high antibody concentrations to enhance the binding kinetics. This is advantageous, as it reduces nonspecific background and further minimizes cross-reactivity.^14^

Due to the apparent benefits, anti-IC antibodies have been developed for various small targets, including morphine,^20^ cyanotoxins,^21^ and estradiol.^22^ More recently, anti-IC antibodies have also been developed for testosterone,^23^ although with unknown analytical performance. *In vitro* display techniques, such as phage display, have facilitated the development process of anti-IC antibodies against these challenging targets. Unlike traditional immunization-based methods, phage display allows precise control over selection conditions, enabling the generation of antibodies against molecular complexes with limited stability.^24,25^

Here, we present the creation of a recombinant anti-IC fragment antigen-binding (Fab), which binds to an anti-testosterone monoclonal antibody in the presence of testosterone, and the use of this antibody pair in the development of a proof-of-concept non-competitive time-resolved fluorescence (TRF) immunoassay for the detection of testosterone (Figure 1A). The anti-IC Fab was generated using a synthetic antibody library in concert with phage display. The developed immunoassay demonstrated reliable detection of testosterone concentrations within the physiological range in adult men and women, as well as children.

**Figure 1:**
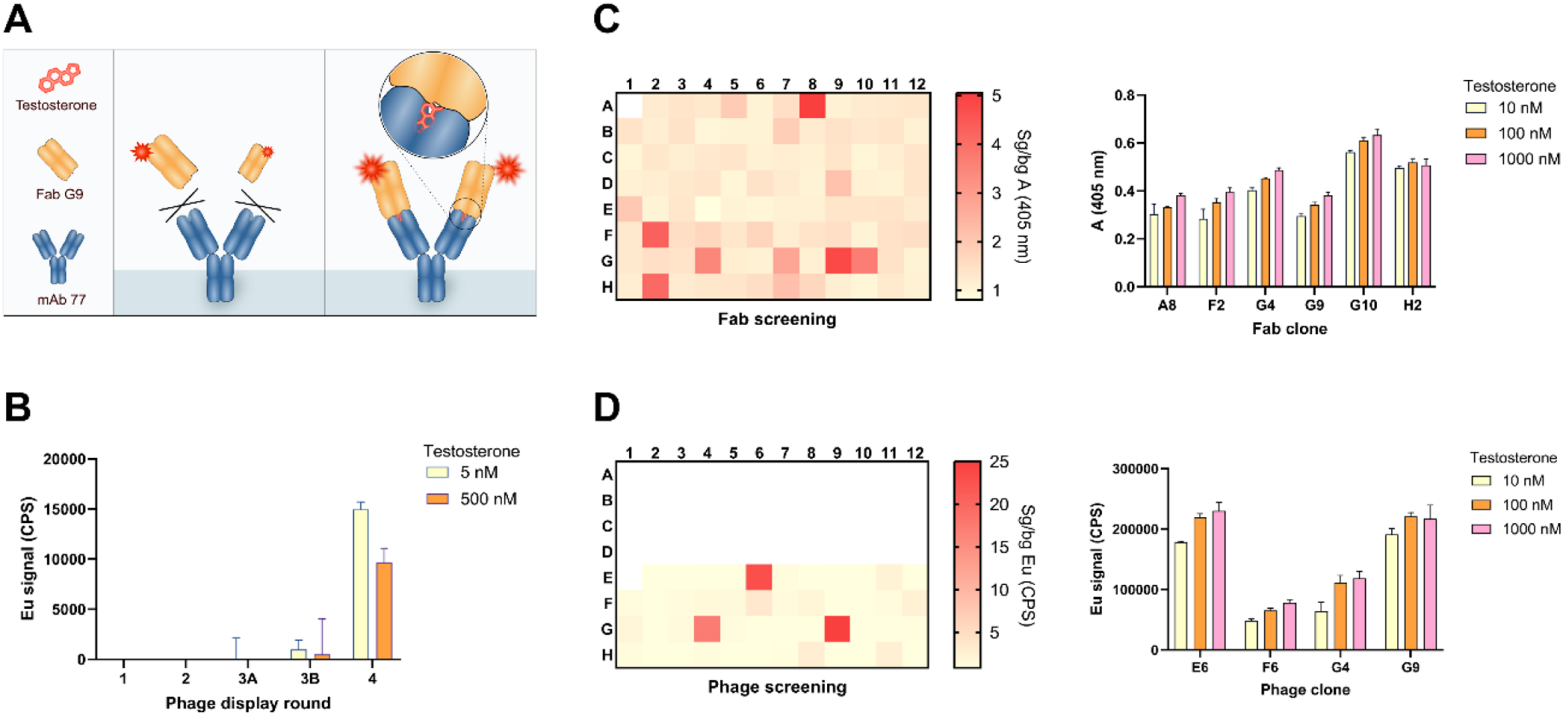
Anti-IC assay concept and screening of testosterone anti-IC phages and Fabs. (A) The anti-IC Fab G9 does not bind mAb 77 in the absence of testosterone. When testosterone is present, Fab G9 binds to the IC formed by mAb 77 and the steroid. (B) Immunoreactivity of phage stocks after each selection round. The immunoreactivity was analyzed using 50 ng of surface-immobilized mAb 77 and 5 and 500 nM testosterone. Primary screening of anti-IC (C) Fabs and (D) phages. The screening was performed in a 96-well plate, where the signal from wells containing the IC (1 μM testosterone) was compared with that from background wells with no testosterone. The heat maps show the signal-to-background ratio for the absorbance at 405 nm and Eu signal, respectively. Empty wells are in white. The best-performing testosterone clones were analyzed further using 10–1000 nM of testosterone. The error bars represent the SD of three replicate measurements.

## Experimental Section

### Reagents and Instrumentation

The anti-testosterone monoclonal antibody 77 (mAb 77) described by Valjakka et al. (2002) was used as the primary antibody in this study. Tosyl-activated paramagnetic beads were from Invitrogen Dynal AS (Norway) and Dynabeads MyOne Streptavidin C1 beads and avidin-coated Dynabeads M-270 Epoxy were from Thermo Fisher Scientific (USA). All hormones were from Sigma-Aldrich (USA). The plate washer was from Revvity (USA), and the assay buffer (50 mM TSA pH 7.75, 0.01% Tween 40, 0.05% bovine-γ-globulin, 20 μM DTPA, 0.5% BSA, 20 μg/mL cherry red) and low-fluorescence streptavidin coated microtiter plates were from Uniogen Oy (Finland). The europium (Eu) chelate N^1^(pisothiocyanatobenzyl)­diethylenetriamine-N,^1^N^2^,N^3^,N^3^-tetraacetic acid and the Europium Enhancement Solution (EES) were from University of Turku (Finland). All Eu and absorbance measurements were done with Hidex Sense (Hidex, Finland). Statistical analyses were done using Prism 10 (GraphPad Software, USA), and figures were illustrated using Affinity Designer 2 (Serif Europe Ltd., UK).

### Phage Display Selections

The anti-testosterone mAb 77 binds testosterone to form the IC, which served as the target structure in both the phage display selections and subsequent immunoassays. Testosterone anti-IC Fabs were enriched from a synthetic Fab library (Biotechnology Unit, University of Turku) containing 1 × 10^12^ phages through phage display, using four rounds of selection under varying conditions.

In rounds 2–4, the phage stock from the previous round was subjected to negative selection before incubation with the target to deplete phages binding to the empty primary antibody. The phage stock was incubated in TBT-0.05 (50 mM Tris pH 7.5, 150 mM NaCl, 1% BSA, 0.05% Tween 20, 0.02% NaN_3_) with the biotinylated primary antibody bound to a solid matrix (microtiter plate or magnetic beads) and incubated at room temperature for 1 h. The unbound fractions were used in the IC selections.

To capture the IC-specific phages, beads with different chemistries were conjugated with mAb 77. In the first selection round, mAb 77 was covalently coupled to Tosyl-activated paramagnetic beads, whereas in the second round the biotinylated antibody was conjugated to Dynabeads MyOne Streptavidin C1. In the third and fourth selection round, biotinylated mAb 77 was conjugated to avidin-coated Dynabeads M-270 Epoxy beads. In the two first rounds of the IC selections, the conjugated beads were simultaneously incubated with testosterone and the phage library. In round 3, two parallel selection conditions were applied. Strategy A was done as described above, whereas in strategy B the beads were first incubated with testosterone for 30 min before excess hormone was removed and the phages were added. Round four was carried out following the conditions of strategy B. An excess of nonspecific estradiol-binding Fab, described by Lamminmäki et al. (2003),^26^ was added as a negative selection in all rounds. The Fab was free in the solution, ensuring that phages specific for generic Fab surfaces were removed in the washing step. The incubations were done at +4 °C, either for 2 h or overnight.

The enrichment of phages specific to the IC was assessed after each round by subjecting the phage library to a TRF immunoreactivity assay. After each step in the assay, incubations were performed at room temperature for 1 h with low shaking, and washed four times using a plate washer. Biotinylated mAb 77 (500 ng) in assay buffer was conjugated to low-fluorescence yellow streptavidin wells and after incubation, testosterone (5 and 500 nM) diluted in assay buffer was added in triplicates. 1.0 × 10^8^ phages from the phage stocks were added and detected with 4 ng of europium-labeled (Eu-labeled) antiphage antibody per well. After a 10 min incubation with 200 μL EES, the TRF Eu signal was measured using a standard Eu protocol with the excitation wavelength 340 nm and the emission wavelength 615 nm (Figure 1B).

### Screening

After the fourth selection round, screening was done in both soluble Fab format and phage format in parallel. The enriched phage library was cloned into an expression vector for production as Fabs fused with bacterial alkaline phosphatase (AP) (Biotechnology Unit, University of Turku). The constructs were transformed into XL1 Blue *E. coli* cells, and colonies (*n = 95*) were picked from the transformation plates. The screening was done using an AP-based enzyme-linked immunosorbent assay (ELISA): After induction with 100 μg/mL IPTG and an overnight incubation at 26°C, the cells were centrifuged and the supernatants containing secreted Fabs were diluted 1:4 in assay buffer. Streptavidin-coated microtiter wells were conjugated with biotinylated mAb 77 (50 ng) and testosterone (10 nM, 100 nM and 1000 nM) was added in triplicates. After incubating shaking at room temperature for 30 min, the wells were washed four times with a plate washer. Thereafter, 100 μL of the Fab-assay buffer mix was added. The wells were incubated at room temperature for 1 h and washed four times. Then, 1 mg/mL pNPP in pNPP buffer (500 mM Tris, 200 mM NaCl, 10 mM MgCl_2_, pH 9) was added and the assay was incubated at +37°C until yellow color was visible. The absorbance of the reactions was measured at 405 nm (Figure 1C). Selected anti-IC Fabs were analyzed further using 10–1000 nM testosterone (Figure 1C).

In parallel, phage colonies (*n = 47*) were screened as described previously^27^ and the supernatants containing the Fab-expressing phages were used for the TRF screening immunoassay. The immunoassay was performed as described for the TRF immunoreactivity assay, with some modifications to the reagent quantities: 50 ng of biotinylated mAb 77, 1 μM testosterone, 50 μL of phages from the screening cultures described in the previous section diluted to 1:4 in assay buffer, and 12.5 ng anti-phage antibody (Figure 1D). Selected anti-IC phages were analyzed further using 10–1000 nM testosterone (Figure 1D).

### TRF Immunoassay

The Fab with the most promising binding properties based on the screening (Fab G9) was labeled with a 50-fold molar excess of the Eu-chelate N^1^(pisothiocyanatobenzyl)­diethylenetriamine-N,^1^N^2^,N^3^,N^3^-tetraacetic acid in 50 mM carbonate buffer (pH 9) using a previously described protocol.^28^ After an overnight incubation at room temperature in the dark, the labeled antibodies were separated from the free chelate by FPLC. The labeling degree was 2 Eu molecules per Fab molecule.

All TRF immunoassays had a similar setup: biotinylated mAb 77 (50–100 ng) was conjugated to streptavidin wells and incubated shaking for 1 h, followed by four washes with a plate washer. Then, the sample or testosterone was added and the reaction was incubated shaking for 1 h. After another wash, Eu-labeled Fab G9 (50–100 ng) was added and incubated shaking for 1 h. The TRF was measured after incubating for 10 min with EES. The final volume in each step was 200 μL and the incubations were done at room temperature.

The EC50 for the TRF immunoassay was determined from a standard curve of 12 different concentration points of testosterone (0–25000 pM) (Figure 2A). The LoD and limit of quantitation (LoQ) were calculated based on the mean of the background +3 × SD and 10 × SD, respectively (Figure 2B).

**Figure 2:**
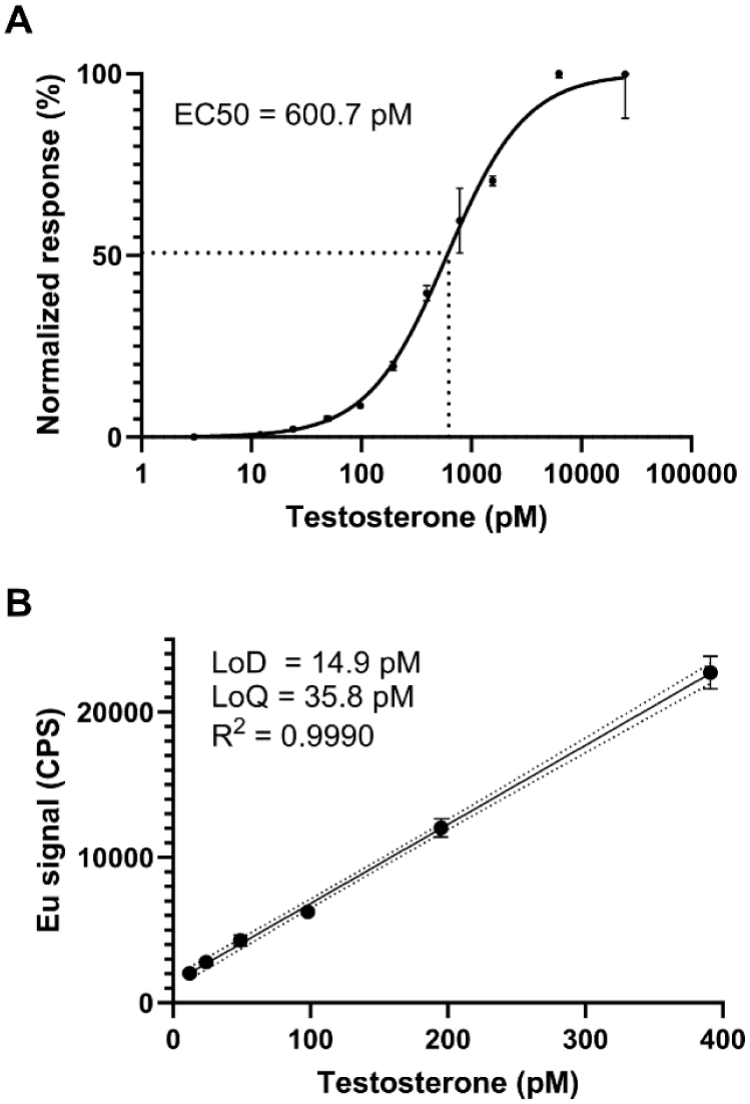
TRF assay performance. (A) The standard curve was generated using 12 concentrations of testosterone ranging from 0 to 25000 pM. The data was normalized setting the lowest value to 0% and the highest value to 100%, and a nonlinear regression was performed to generate the standard curve. (B) The LoD was calculated as the mean of the background +3 × SD and the LoQ was calculated as the mean of the background +10 × SD. The LoD was interpolated from the dynamic range of the standard curve using a straight curve fit. The error bars represent the SD of three replicate measurements.

The specificity of the immunoassay was evaluated using testosterone as the reference analyte. Structurally related and circulation-abundant steroid hormones—11-keto testosterone, 5α-dihydrotestosterone (DHT), androstenedione, estradiol, and cortisol—were evaluated for cross-reactivity at final concentrations of 0.1 nM, 100 nM, and 1000 nM. All samples, including testosterone, were prepared in assay buffer and analyzed under identical conditions (Figure S1). To evaluate the cross-reactivity profiles for DHT, 11-keto testosterone and androstenedione in more detail, concentrations within and beyond the physiological range of the steroids were titrated to wells containing a constant concentration of testosterone (600 pM) (Figures S2–S4).

### Matrix Effect on TRF Immunoassay

Plasma and serum samples were collected with informed consent from healthy male and female donors from the lab members of the Biotechnology Unit at University of Turku. To reduce the effect of endogenous testosterone in the experiments using these samples, steroid hormones were removed following the method described in Sikora et al. (2016). Briefly, the samples were incubated with dextran-coated charcoal rotating at +4°C for 12 h, whereafter the charcoal was pelleted by centrifugation at +4°C for 15 min at 10 000 g and the sample was filtered with a 0.22 μm filter.

The effect of the sample matrix on the assay performance was analyzed using separate pools of charcoal-stripped serum and plasma samples. The samples were diluted to 2.5, 5, 10, 20, and 40% in assay buffer and spiked with 100–1000 pM testosterone in triplicates (Table 1). Next, three individual charcoal-stripped plasma samples were spiked with 50–800 pM testosterone and the recovery capability of the assay was analyzed. The specific signals were compared to a standard curve of 0–8000 pM testosterone in assay buffer (Table S1).

**Table 1:** Matrix Effect of the TRF Immunoassay

		Plasma	Serum
Testosterone (pM)	Matrix (%)	Signal (%)	Signal (%)
100	0	100	100
	2.5	133	124
	5	115	107
	10	85	120
	20	100	255
	40	110	0
500	0	100	100
	2.5	108	96
	5	100	94
	10	80	66
	20	63	58
	40	40	15
1000	0	100	100
	2.5	96	88
	5	76	89
	10	72	65
	20	63	47
	40	57	28

Three individual charcoal-stripped plasma samples were analyzed on two consecutive days to assess the variability and total imprecision of the assay. The samples were diluted to 20% and spiked with 100–1000 pM testosterone (Table 2). The intra-assay variability was calculated as the average of the CV% for the different samples at the same testosterone concentration analyzed in the same assay run. The inter-assay variability was calculated from the averages of CV% of the samples at the same testosterone concentration, run on different days. The total imprecision was calculated as the sum of the inter- and intra-assay variability.

**Table 2:** Intra-Assay, Inter-Assay, and Total Imprecision of the Immunoassay Using Three Individual Samples Spiked with 100–1000 pM Testosterone

	CV%		
Testosterone (pM)	Intra-assay (*n* = 3)	Inter-assay (*n* = 3 × 2)	Total
100	5.1	7.0	12.1
500	6.0	4.9	10.9
1000	3.1	4.3	7.4

## Results and Discussion

### Antibody Development

In this study, we used a highly diverse synthetic antibody phage library to enrich anti-IC binders recognizing the recombinant mAb 77 bound to testosterone. To guide the selection toward the interface of mAb 77 and testosterone as a complex, binders with unwanted specificities were depleted from the phage pool by selection against the free mAb 77 prior to exposing them to the target IC. In addition, an excess of estradiol-specific Fab fragment was included for counter-selection in the solution. The stringency was gradually increased each round by reducing the amount of mAb 77 in the reaction and by increasing the number of washing steps. In round 3, the two parallel selection conditions yielded different results. Enrichment of IC-specific phages was observed with strategy B where excess testosterone was removed before the phage library was added (Figure 1B), and this strategy was therefore also used in round 4.

The primary screening was done in a Fab (Figure 1C) and a phage format (Figure 1D), in parallel. The clones that showed a binding response in the presence of 1 μM testosterone were analyzed further using three different concentrations of testosterone (10–1000 nM). Sanger sequencing showed that clone G10 from the Fab screening, and clones E6 and G9 from the phage screening were identical. The recurrence of the same clone across independent screening pathways provides further evidence of successful enrichment of the phage library and implies that this particular Fab clone possesses favorable binding characteristics.

### TRF Immunoassay

Next, the primary mAb 77 and purified anti-IC Fab G9 were used to set up a non-competitive TRF immunoassay for the detection of testosterone. After optimizing the reagent concentrations, a standard curve for the immunoassay was generated using 12 concentration points of testosterone ranging from 0 to 25000 pM (Figure 2A). As deduced from the standard curve, the EC50 for the assay was 600.7 pM, while the LoD was 14.9 pM, and the LoQ was 35.8 pM (Figure 2B). Given that serum testosterone levels range from 10 to 38 nM in men, 0.4 to 2 nM in women, and 2 to 13 nM in children, the assay demonstrates adequate analytical performance for the quantification of circulatory testosterone levels in all groups.

Based on our previous experience with other small molecule analytes, the performance of anti-IC immunoassays depends largely on the properties of the primary antibody.^22^ The primary antibody used in this study, mAb 77, in many ways provides a good starting point for the anti-IC assay development. First, it is known to bind testosterone with high affinity (K_d_ = 0.3 nM). Moreover, it exhibits low cross-reactivity with the structurally similar molecules DHT, dehydroepiandrosterone sulfate, and androstenedione (<10%). Of the three steroids, the cross-reactivity for DHT is the highest (7.9%).^29^

Anti-IC antibodies have been reported to stabilize the binding of the hapten to the primary antibody, thereby further improving assay specificity.^30^ To evaluate this effect in our TRF assay, the potential cross-reactivity was tested against the structurally similar and circulation-abundant steroids 11-keto testosterone, DHT, androstenedione, estradiol, and cortisol in buffer-based matrix (Figure S1). No cross-reactivity was observed for estradiol and cortisol. DHT and androstenedione, which are known to cross-react slightly with the primary antibody mAb 77, caused a signal increase in the higher concentrations. The cross-reactivity of the primary antibody toward 11-keto testosterone had not been evaluated in the original publication,^29^ and in the present anti-IC assay, significant cross-reactivity was observed already in the lowest concentration. 11-keto testosterone is a testosterone metabolite with high androgen potency,^31^ with reported serum concentrations of up to 1.7 nM in healthy individuals and as high as 12 nM in patients with congenital adrenal hyperplasia caused by 21-hydroxylase deficiency.^32^ To evaluate the effect of 11-keto testosterone, DHT, and androstenedione on assay performance in the presence of testosterone, the steroids were each titrated to a constant concentration of testosterone (600 pM). The titration series included concentrations both within and beyond the physiological range of each steroid. Adding 11-keto testosterone to the reaction increased the signal level when reaching nanomolar concentrations, indicating that the steroid binds to the primary antibody without blocking the anti-IC antibody from binding (Figure S2). Interestingly, given the cross-reactivity profile of mAb 77, the addition of DHT (Figure S3) and androstenedione (Figure S4) caused no substantial change in signal. Together, these findings suggest that the anti-IC antibody reduces the cross-reactivity with DHT and androstenedione by stabilizing the testosterone–mAb 77 complex in the immunoassay.

### Matrix Effect on Analytical Performance of the TRF Immunoassay

Next, to assess the effect of sample matrix on the performance of the immunoassay, we used pools of plasma and serum, respectively, from healthy male and female donors. Signals from wells containing no testosterone were subtracted from those with testosterone to account for background effects caused by endogenous testosterone in the samples. Assay signals obtained in plasma and serum were compared to signals from buffer-only wells. The mean signal of buffer wells was set to 100%, and signals from wells containing increasing concentrations of plasma or serum were expressed relative to this reference. Each matrix concentration was spiked with three testosterone concentrations (100–1000 pM) to evaluate the assay performance across a wider range. At 100 pM of testosterone, the matrix generally caused an increase in signal, bringing it above 100%. This is a reflection of the limitations of the analytical performance at the lower range of the assay, which can also be observed in the overall variability at the same concentration (Table 2). At 500 pM, which is close to the EC50 of the immunoassay, the increasing concentrations of plasma resulted in less changes to the signal levels than serum. The same could be observed for the signals at 1000 pM (Table 1). Consequently, only plasma samples were used in the subsequent experiments. We continued with the highest reliable matrix concentration (20%) to maximize the proportion of sample that can be used in future applications.

With further optimization of the assay conditions, a similar approach could be adapted for analysis of other sample matrices such as saliva and urine, which would broaden the applicability to noninvasive testosterone monitoring. This also opens up the possibilities for homogeneous anti-IC assay formats, such as the FRET assay, which have previously been reported for other small molecule analytes.^13,15,33^

The intra-assay, inter-assay, and total imprecision of the immunoassay were assessed by analyzing three individual plasma samples spiked with 100, 500, and 1000 pM testosterone on two consecutive days (Table 2). The low intra- and inter-assay CVs observed across the tested testosterone concentrations demonstrate good repeatability and reproducibility of the assay, while the total imprecision values indicate that the assay performs reliably across the physiologically relevant range of testosterone. To analyze the recovery capability of the immunoassay, three individual charcoal-stripped plasma samples were spiked with variable amounts of testosterone (Table S1). Higher recoveries were observed in the male samples, likely due to incomplete removal of endogenous testosterone. In the lower range of the spiked samples, the low recovery percentages may be explained by unoccupied sex hormone-binding globulin (SHBG) in the charcoal-stripped samples binding the added testosterone and thereby reducing the measurable recovery. Recovery improved at higher testosterone concentrations, consistent with saturation of the remaining binding capacity.

## Conclusions

In this work, we present the development of a novel anti-IC antibody that specifically binds to a testosterone–antibody IC and demonstrate its use in a proof-of-concept non-competitive TRF immunoassay for the detection of testosterone. To our knowledge, this is the first non-competitive anti-IC immunoassay for testosterone. The development of alternative assay concepts addresses a longstanding issue identified by The Endocrine Society, which over a decade ago highlighted the limitations of existing testosterone immunoassays in terms of accuracy, cost and reliability. The anti-IC assay has the potential to overcome the sensitivity challenges often associated with small-molecule immunoassays, thereby offering a promising alternative to conventional competitive formats.

To ensure future clinical reliability, the immunoassay requires validation in larger and more diverse cohorts where testosterone concentrations have been established using reference methods. A direct comparison with LC-MS/MS and currently available immunoassays will be essential to assess the performance and clinical applicability. In addition, the interference of serum 11-keto testosterone on the testosterone measurements needs to be studied in the future and addressed in a way that ensures testosterone specificity. Alternatively, the assay could be adapted to extend its detection profile toward assessing androgen potency.

This study highlights the potential of phage display technology for generating antibodies with unique specificity and establishes a foundation for future improvements in testosterone detection. Moreover, the anti-IC approach provides flexibility for translation into other assay formats, including homogeneous platforms, which can further reduce assay time and simplify implementation in clinical laboratories.

## Supplementary Material



## Notes

Experimental data of cross-reactivity and recovery (PDF)
