# Supplementary material for: Development of an Anti-Immunocomplex Antibody and Non-competitive Immunoassay for the Detection of Testosterone
Source: Anal Chem. 2026 Feb 26;98(9):6620–6. doi: 10.1021/acs.analchem.5c06003 (PMC12980484; doi:10.1021/acs.analchem.5c06003)
Supplement: Supplementary file 1 [file ac5c06003_si_001.pdf]

Supporting Information

Development of an anti-immunocomplex antibody and non-competitive immunoassay for the detection of testosterone

Ida Bäckström[1*], Urpo Lamminmäki[1], Etvi Juntunen[2], Janne Leivo[1,3]

[1]Department of Life Technologies and InFLAMES Flagship, University of Turku, 20014 Turku, Finland
[2]Olo Health Oy, 20100 Turku, Finland
[3]FICAN West Cancer Centre Laboratory, University of Turku and Turku University Hospital, 20014 Turku, Finland
* Corresponding author, email: isback@utu.fi

Table of Contents

Figure S1. Specificity of the TR-F assay S2

Figure S2. Titration of 11-keto testosterone to the TR-F assay S2

Figure S3. Titration of DHT to the TR-F assay S3

Figure S4. Titration of androstenedione to the TR-F assay S3

Table S1. Recovery of testosterone from spiked plasma samples S4

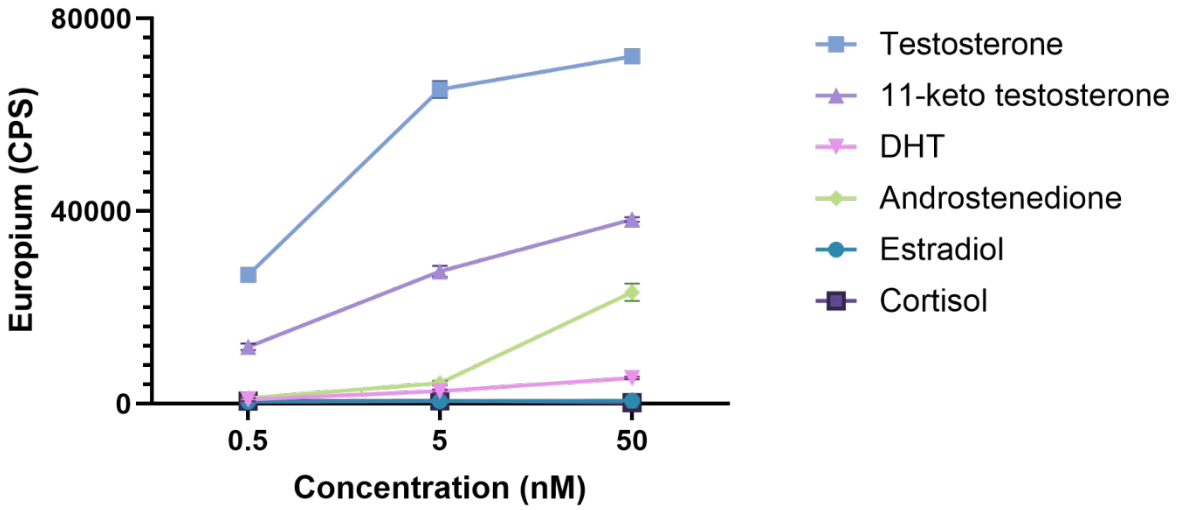

Figure S1. Specificity of the TR-F assay. The cross-reactivity was evaluated against the structurally similar and circulation-abundant steroids 11-keto testosterone, DHT, androstenedione, estradiol, and cortisol. Signals from wells with only buffer have been subtracted from those containing steroids. The error bars represent the SD of three replicate measurements.

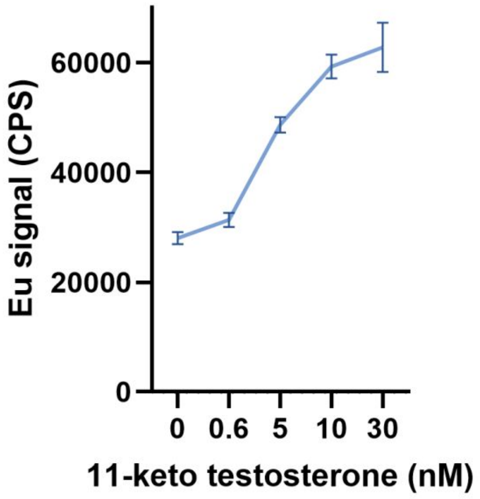

Figure S2. Titration of 11-keto testosterone to the TR-F assay. 11-keto testosterone was titrated to a constant concentration of testosterone (600 pM). The error bars represent the SD of three replicate measurements.

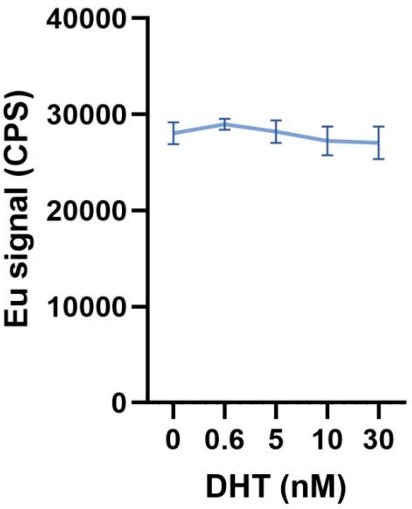

Figure S3. Titration of DHT to the TR-F assay. DHT was titrated to a constant concentration of testosterone (600 pM). The error bars represent the SD of three replicate measurements.

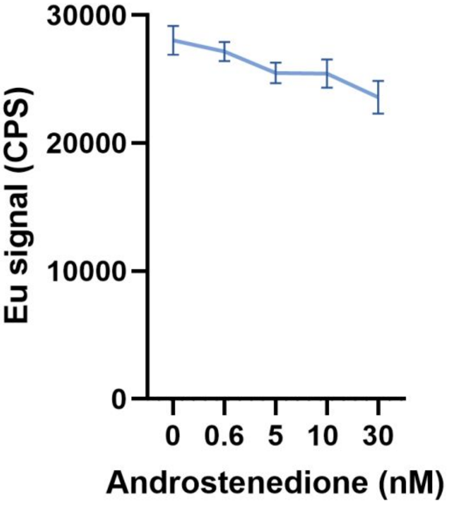

Figure S4. Titration of androstenedione to the TR-F assay. DHT was titrated to a constant concentration of testosterone (600 pM). The error bars represent the SD of three replicate measurements.

Table S1. Recovery of testosterone from spiked plasma samples. The recovery capability of the testosterone TR-F immunoassay was assessed using tree charcoal-stripped plasma samples spiked with 50–800 pM testosterone.

**Testosterone (pM)**	**Plasma**					
	Sample 1 (female)		Sample 2 (male)		Sample 3 (male)	
	Recovery (%)	CV%	Recovery (%)	CV%	Recovery (%)	CV%
**50**	0	4.3	47	7.9	47	3.3
**100**	0	3.7	49	5.5	48	5.7
**200**	30	7.4	79	0.9	92	12.9
**400**	23	2.8	95	1.6	64	4.2
**800**	46	3.5	115	5.4	72	4.8
